# Expanding the Natural History of *SNORD118*-Related Ribosomopathy: Hints from an Early-Diagnosed Patient with Leukoencephalopathy with Calcifications and Cysts and Overview of the Literature

**DOI:** 10.3390/genes14091817

**Published:** 2023-09-19

**Authors:** Davide Politano, Guido Catalano, Elena Pezzotti, Costanza Varesio, Fabio Sirchia, Antonella Casella, Elisa Rognone, Anna Pichiecchio, Renato Borgatti, Simona Orcesi

**Affiliations:** 1 Department of Brain and Behavior Neuroscience, University of Pavia, 27100 Pavia, Italy; 2 Child Neurology and Psychiatry Unit, IRCCS Mondino Foundation, 27100 Pavia, Italy; 3 U.O. Neuropsichiatria Infanzia e Adolescenza, ASST Bergamo Est, 24068 Seriate, Italy; 4 Department of Molecular Medicine, University of Pavia, 27100 Pavia, Italy; 5 Medical Genetics Unit, IRCCS San Matteo Foundation, 27100 Pavia, Italy; 6 Neurogenetics Research Center, IRCCS Mondino Foundation, 27100 Pavia, Italy; 7 Neuroradiology Department, IRCCS Mondino Foundation, 27100 Pavia, Italy

**Keywords:** *SNORD118*, coats plus, LCC, bevacizumab, ribosomopathy, natural history

## Abstract

Leukoencephalopathy with calcifications and cysts (LCC) is a rare autosomal recessive disorder showing a pediatric or adult onset. First described in 1996 by Labrune and colleagues, it was only in 2016 that bi-allelic variants in a non-protein coding gene, *SNORD118*, were found as the cause for LCC, differentiating this syndrome from coats plus (CP). *SNORD118* transcribes for a small nucleolar RNA, which is necessary for correct ribosome biogenesis, hence the classification of LCC among ribosomopathies. The syndrome is characterized by a combination of white matter hyperintensities, calcifications, and cysts on brain MRI with varying neurological signs. Corticosteroids, surgery, and recently bevacizumab, have been tried with unclear results since the natural history of the disease remains elusive. To date, 67 patients with a pediatric onset of disease have been described in the literature, with a clinical-radiological follow-up carried out in only eleven of them. We described the clinical-radiological follow-up from birth to almost five years of age of a late-preterm patient diagnosed with LCC and carried out a thorough overview of pediatric patients described in the literature. It is important to gather serial clinical–radiological data from other patients to depict the natural history of this disease, aiming to deeply depict genotype-phenotype correlations and make the role of new therapeutics clearer.

## 1. Introduction

Leukoencephalopathy with calcifications and cysts (LCC) is a rare autosomal recessive neurological disorder. It has an estimated prevalence of less than one in a million individuals, with only approximately a hundred cases documented in the existing literature up to this point [1,2,3,4].

In 1996, Labrune and colleagues clinically characterized LCC in three unrelated individuals born from non-consanguineous parents, hence the name Labrune syndrome. These individuals exhibited cognitive decline, rare epileptic seizures, and various neurological signs with clinical onset ranging from early childhood to adolescence. Their brain imaging showed white matter signal hyperintensities on T2-weighted imaging, progressive brain calcifications, and cyst development [5]. The absence of cerebral atrophy is a useful radiological discriminator [2].

Eight years earlier, in 1988, Tolmie and colleagues described two sisters affected by bilateral exudative retinopathy, also known as Coats disease, associated with various neurological and extra-neurological findings (gastrointestinal, skeletal, and ectodermic disturbances) with neuroradiological alterations mirroring those of LCC, which he then termed Coats Plus (CP) syndrome [6,7].

Given the neuroradiological similarities, LCC and CP were considered as two entities within the same spectrum of disorders until 2012, when it was discovered that bi-allelic pathogenic variants in CTC1 were found exclusively in patients affected by CP [8], while absent in LCC patients. Subsequently, in 2016, Jenkinson and colleagues identified bi-allelic variants in *SNORD118*, encoding for a small nucleolar RNA (snoRNA) known as U8 snoRNA, as the genetic cause for LCC [9].

U8 snoRNA is involved in the maturation of the 60S ribosome subunit [10]. Microangiopathic changes manifest as heightened tortuosity and calcification in small blood vessels, resulting in augmented focal edema and increased water content in the white matter [9,11,12,13,14]. Nevertheless, even though U8 snoRNA plays a pivotal role in ribosomal biology, the pathophysiological connection between a potentially global disturbance in ribosomal function and the development of cerebral microangiopathy with highly stereotyped radiological characteristics remains unclear [1].

Apart from the typical neuroradiological triad consisting of white matter hyperintensities on T2-weighted imaging, cerebral calcifications, and parenchymal cysts, clinically, LCC is often characterized by epilepsy, accompanied by global developmental delay and sometimes followed by slow regression. Additional neurological symptoms vary, partly depending on the location and volume increase of the cysts, with some cases requiring cyst drainage due to intracranial hypertension and hydrocephalus [15]. Disease onset can occur in pediatric and adult populations, with no clear genotype–phenotype correlation [1]. From the therapeutic point of view, corticosteroid treatment and surgical procedures have been performed with sometimes partial and temporary benefits [16,17].

Given the microvascular alterations observed in brain biopsies, the anti-angiogenic agent bevacizumab has been administered to some LCC patients in recent years; however, its effectiveness remains uncertain since the natural history of the disease is unknown due to either the description of isolated cases or the lack of standardized serial evaluations from a clinical-radiological standpoint for prominent cohorts of patients [11,15,16].

To date, less than a hundred patients have received a clinical and genetic diagnosis of LCC with symptoms starting before 18 years of age. Follow-up evaluations of neuroradiological and clinical characteristics were reported in only in few pediatric cases, often with short-term follow-up and the absence of standardized clinical evaluations.

This article aims to provide a detailed description of the clinical and neuroradiological picture of a late-preterm patient with an early diagnosis of LCC and his clinical-neuroradiological follow-up from birth to five years of age, hence documenting the early natural history of disease of our almost asymptomatic case.

## 2. Materials and Methods

After obtaining written informed consent from legal guardians, clinical data were acquired from the proband’s medical records related to his hospitalizations at the IRCCS Mondino Foundation (Pavia, Italy). The studies involving human participants were reviewed and approved by the Local ethics committee (approval n.: 0099934/21; 8 November 2021). Clinical and neurological evaluations were performed. Moreover, a specific age-appropriate neuropsychological assessment was administered (Griffith’s Scale of Child Development 3rd edition). The patient underwent 3 Tesla (3T) brain MRIs (Magnetom Skyra, Siemens Healthcare, Erlangen, Germany) with and without sedation using multiplanar T1- and T2-weighted images with age-appropriate TR and TE values. A Sanger sequencing on genomic DNA was performed.

## 3. Results

Proband is a 4-year-and-6-month-old male patient, the first child of unrelated parents, referred from birth to our outpatient clinic for prematurity follow-up. No family history of neurological, autoimmune, or vascular diseases was reported.

Pregnancy was uneventful, and delivery occurred at 34 weeks and two days of gestation by an urgent cesarean section due to premature rupture of membranes and associated signs of fetal distress. Auxological parameters at birth were within normal limits: birth weight 2180 g (25–50 °p), length 44 cm (25–50 °p), and head circumference 30 cm (10–25 °p). Apgar index was 7 and 9, respectively, at the 1st and 5th minute. At birth, the patient presented signs of mild respiratory distress and needed oxygen therapy. Serial cerebral ultrasounds (cUS) documented diffuse white matter hyperechogenicity, mild hyperechogenicity of the basal nuclei, and bilateral thalami associated with lenticulostriate vasculopathy. Intrauterine infection by a TORCH agent was ruled out: maternal serologies were unremarkable during pregnancy, and CMV-RNA tested negative on newborn urine at birth. Neurological examination at term showed some hyperexcitability signs and poor spontaneous motility. Echocardiography showed minimal patent foramen ovale; eye examination and abdominal ultrasound were within normal limits.

**Figure 1:**
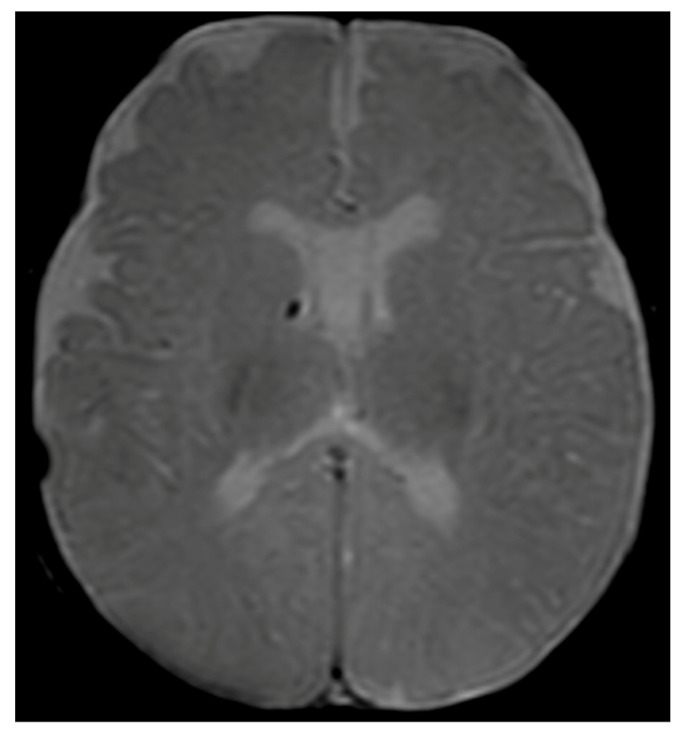
Brain MRI with axial T2-gradient echo image at 2 months and 3 weeks of life. Punctate susceptibility focus, suspicious for calcification/hemosiderin, in the right germinal matrix is visible.

Considering the persistence of pathological signs on cuS, a brain MRI was performed at two months and three weeks of corrected age (CA), showing a hypointense nodular alteration through gradient echo weighted images (Figure 1).

Over the next months, the infant presented idiopathic (antibody-negative) neutropenia with subsequent spontaneous resolution.

At 6 months CA, the patient showed slight developmental delay. He could sit unsupported at 8 months CA and walk independently at 14 months CA. At 20 months CA, a slight delay in expressive language was noted; however, language comprehension and communicative purpose were normal for age. 

**Figure 2:**
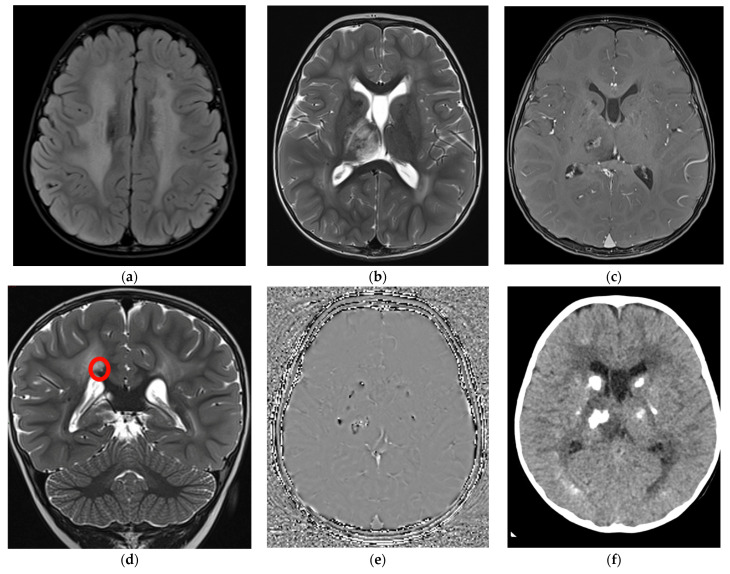
Brain MRI at 2 years and 3 months with axial Flair (**a**), axial (**b**), and coronal (**d**) T2-TSE, axial T1-Tse post gadolinium (**c**), phase image of the susceptibility weighted imaging (SWI) (**e**), and axial CT without contrast (**f**). (**a**) a diffuse white matter hyperintensity is evident in both hemispheres; (**b**,**c**) a right thalamic involvement is also evident with central and not homogeneous contrast enhancement after gadolinium injection; (**d**) a small millimetric cyst (red circle) can be detected over the right portion of the splenium of the corpus callosum, hyperintense on T2; (**e**) diffuse calcification are documented as hypointense foci on SWI (**e**) and hyperdense on CT (**f**) in both basal ganglia, thalami were they appear as rock lesions and juxtacortical, where they are more punctate or linear.

At two years and three months of age, mild neuropsychomotor delay was confirmed through the Griffiths Scale of Child Development 3rd edition (developmental quotient was 70, at the 2nd percentile) with greater impairment in expressive language (language and communication quotient 65 that is under the 1st percentile). At the same age, the head circumference settled at the 50th percentile. Visual and auditory evoked potentials, abdominal ultrasound, ophthalmological examination, and biochemical and metabolic blood assessment were within normal limits. Electroencephalogram in partial sleep deprivation disclosed nonspecific abnormalities (slow and sharp activity in bilateral fronto-central regions) without clinical correlates. Brain MRI scans (Figure 2a–e) documented signs of diffuse leukoencephalopathy with symmetrical white matter involvement with sparing of the corpus callosum and “U” fibers. This finding came with punctate to nodular alterations, confirmed to be calcifications at the subsequent CT scan (Figure 2f), which were predominantly located at the level of the basal nuclei and thalami, cortico-subcortical, and periventricular areas. A one-centimeter cyst was found at the level of the splenium of the corpus callosum.

Evidence of a focal edematous alteration demarcated by contrast enhancement and containing a poorly defined calcification was noted in the right thalamus; this latter image was interpreted as the site of probable future cystic degeneration. The interferon signature result was negative. These elements, associated with the specificity of the neuroradiological features (diffuse leukoencephalopathy with symmetrical white matter involvement, presence of the small intracerebral cyst associated with the right thalamic lesion with a likely cystic evolution and bilateral calcification) and the significant discrepancy between the latter and the mild child’s neurological picture suggested the hypothesis of LCC. Two pathogenic variants in *SNORD118*, n.59T > C and n.*5C > G, were detected at Sanger sequencing, and consequently, the diagnosis of LCC was confirmed.

**Figure 3:**
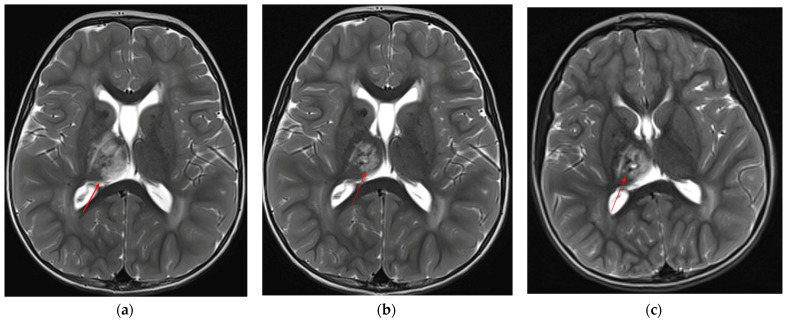
Axial T2-TSE showing progressive reduction and cystic degeneration of the right thalamic lesion (red arrows): (**a**) 2 years and 4 months of age, (**b**) almost 3 years, (**c**) last MRI at almost 5 years of age.

To assess the evolution of the neuroradiological picture and to decide whether to propose an off-label therapy with bevacizumab, the child underwent neuroradiological follow-up at 6-month intervals through brain MRI, which documented spontaneous dimensional reduction of the known signal alteration at the right mesial thalamic site and stability of the leukoencephalopathy (Figure 3a–c).

**Figure 4:**
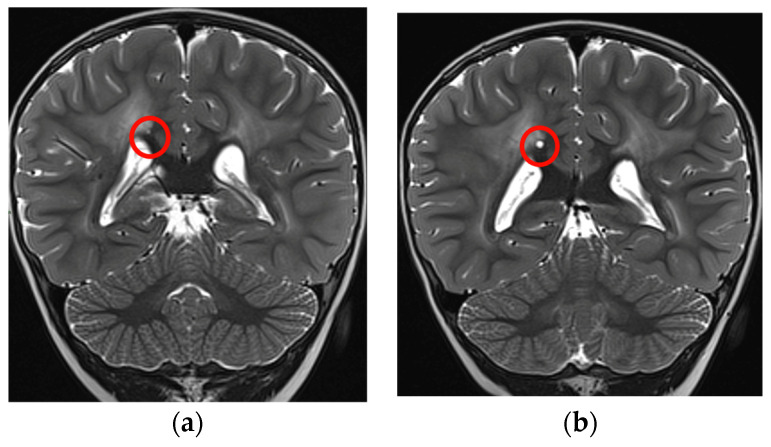
Coronal T2-TSE showing a slight increase in dimension of the cyst (red circles): (**a**) 2 years and 4 months of age, (**b**) almost 3 years.

Moreover, there was an intercurrent millimetric dimensional increase of the small cystic formation along the right margin of the splenium of the corpus callosum that showed a subsequent reduction at the last brain MRI, performed without sedation with a “quick MRI protocol” (Figure 4a,b).

The child has been running psychomotor rehabilitation sessions and regular child neuropsychiatric visits, confirming good and improving psychomotor development. However, he still shows a slightly reduced developmental quotient (78, 7th percentile) with poor expressive language abilities.

In Table 1, a literature overview of pediatric-onset genetically confirmed LCC cases is displayed: 67 patients have received a clinical and genetic diagnosis of LCC with symptoms starting before eighteen years of age, but follow-up evaluations of neuroradiological and clinical characteristics were reported in only eleven of them, with often only short-term follow-up and absence of standardized clinical evaluations.

Based on our comprehensive analysis, seizures emerge as the predominant clinical manifestation in pediatric patients, being reported in nearly half of the cases. This is followed by developmental delay and motor disorders, each described in almost a quarter of cases. Raised intracranial pressure is documented in five patients, while intellectual disability and chronic headaches are each reported in individual cases.

**Figure 5:**
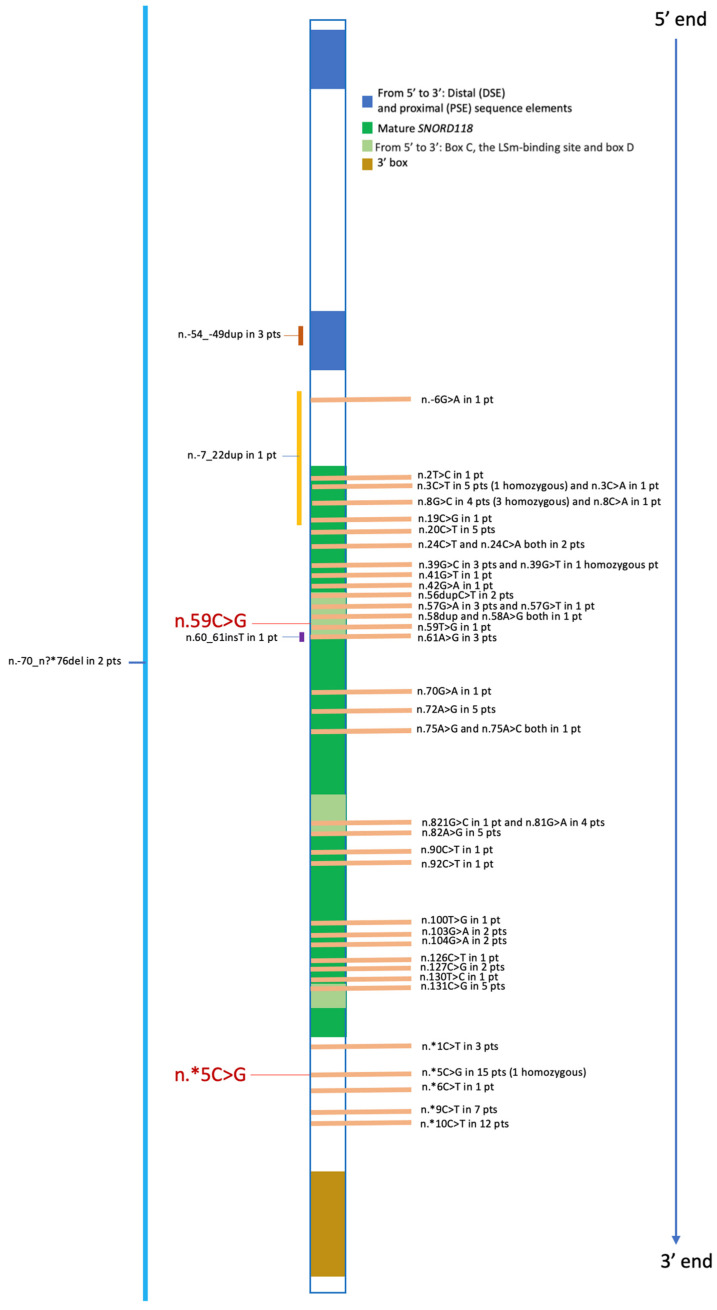
Summary of Pediatric Mutations Reported to Date. For each variant, we provide the count of patients with that specific variant, including those in the homozygous state. Deletions, duplications, and insertions are indicated to the left of the central gene representation bar. The two variants found in our patient are highlighted in red and enlarged. pt, patient; pts, patients.

In Figure 5, all *SNORD118* mutations from our overview of pediatric patients affected by LCC are displayed, focusing on their positioning within the functional and structural domains, including 5′- and 3′-ends.

## 4. Discussion

LCC, classified as a leuko-vasculopathy according to the van der Knaap classification [29], is a rare leukodystrophy characterized by a neuroradiological triad: intracranial calcifications, intraparenchymal cysts, and leukodystrophy. The extent of leukodystrophy and calcification does not appear to correlate with disease severity or time of symptom onset [15]. The localization and size of the cysts, which can enlarge as the disease progresses, do correlate with certain clinical signs and symptoms, sometimes necessitating invasive therapy such as cyst drainage, even urgently. Clinical presentations vary widely, with disease onset occurring from soon after birth to as late as 60 years or older. Symptoms range from progressive cerebellar or pyramidal signs to altered cognitive function, seizures, and even acute neurological symptoms [9] with a nonspecific clinical presentation, as for other leukoencephalopathies [30,31]. Other neurological symptoms can arise, some due to increased intracranial pressure, including headaches and vomiting, while others depend on the locations of the cysts, which often expand in volume. Intractable epileptic seizures with delayed psychomotor development are the most common clinical presentation. Progressive neurological signs manifest as focal motor, sensory, and visual deficits, movement disorders of extrapyramidal and cerebellar origin, and aphasia, all linked to cyst location. Some patients develop signs of increased intracranial pressure due to cyst enlargement [15]. LCC syndrome typically presents as an isolated central nervous system disorder, with only one reported exception involving systemic involvement [17].

The primary cause of this condition is compound heterozygous bi-allelic variants in the ubiquitous *SNORD118* gene, as described by Jenkinson et al. SNORD118, found on chromosome 17p13.1, encodes the U8 snoRNA, a vertebrate-specific small nucleolar RNA responsible for the maturation of the 60S ribosomal subunit. This process involves removing the 3′-external transcribed spacer (3′-ETS) sequence from 28S and 5.8S rRNAs, a critical step in liberating rRNA sequences from the polycistronic precursor-rRNA [32]. Consequently, LCC is classified as a form of Ribosomopathy [33].

Compound heterozygosity is the main transmission mechanism, being the disease enriched in the non-consanguineous family [9]. A complete loss of function (LoF) variant must be associated with a hypomorphic variant to generate LCC: two hypomorphic variants are considered insufficient to give rise to the disease phenotype, while two complete LoF variants might lead to intrauterine fetal death [9,33].

Our patient carries two compound heterozygous pathogenic variants in *SNORD118*: the n.59T > C variant, of maternal origin, which falls in the transcribed region of the gene, has never been described to date although a variant at the same nucleotide level is described in the literature [9] and the n.*5C > G variant, inherited from his father and falling in the 3′ end of the gene, already described in other patients in the literature [1,9,19]. Both variants exhibit low allele frequencies in genome databases of healthy controls, including 1000 Genomes, ExAC, and gnomAD. These variants can potentially disrupt the function and processing of the SNORD118 transcript.

The detailed investigation of the U8 snoRNA structure and its interaction network, as extensively documented by Zhang et al. [34], holds paramount importance when considering the potential pathogenic consequences of variants. The first variant, denoted as n.59T > C, is highly likely to result in a complete loss of function (LOF). This variant occurs at a nucleotide that is vital for the formation of stem-loop (SL) structures, particularly SL3 and SL4 [34], and is situated within the Box C domain of the mature SNORD118, which plays a crucial role in the binding of U8 snoRNA to four key proteins when combined with the Box D domain, namely 15.5 K, and subsequently fibrillarin, NOP56, and NOP58, with facilitation of RNA stabilization, processing, and trafficking.

On the other hand, the second variant is postulated to be hypomorphic in nature, especially considering the existence of reported homozygous cases involving this latter variant [1]. Variants located in the 3′-end alter RNA processing since 5′-end and 3′-end associated during prefolding and subsequent mature RNA conformation and function with plausible alteration in the RNA interaction network [33,34].

As for the clinical history, our patient presented with a mild developmental delay characterized by a prevalent expressive language alteration with clinical improvements during serial follow-up visits. During the first year of life, since he was a late preterm and he showed cUS nonspecific alterations, it was initially assumed that his slight clinical problems could be sequelae of prematurity, but MRI showed the typical neuroradiological triad of disease (intracranial calcifications, intraparenchymal cysts, and leukodystrophy) and suggested the correct diagnosis. White matter changes remained stable, while some evolution emerged during serial follow-up MRI scans, such as dimensional reduction of the right thalamic signal alteration with concurrent increase and subsequent reduction of the millimetric callosal cyst. However, he showed no signs of progression, neither from a clinical nor a radiological standpoint.

Currently, there are no approved treatments available for LCC. Considering the microvascular pathology and the need for a personalized approach like that applied to other deeply documented diseases [35], bevacizumab was introduced as a therapeutic approach for the first adult patient with this leukoencephalopathy. This patient’s condition had its onset during childhood and was initially reported in 2017 by Fay et al. [19]. The patient was born prematurely and exhibited seizures and developmental delays in the first few months of life, likely associated with his premature birth. The first brain MRI was taken at 6 years of age because new neurological signs appeared. It was repeated during the disease course and after treatment with reported consequent radiological and neurological improvement. Bevacizumab, a recombinant humanized monoclonal antibody already employed in treating specific cancer types [36], exerts its anti-angiogenic effects by inhibiting the binding of vascular endothelial growth factor (VEGF) to its receptor. Subsequently, two more LCC patients received bevacizumab, resulting in mixed outcomes: a pediatric patient showed neuroradiological improvement without a concomitant enhancement in clinical status [24], while an adult patient experienced clinical improvement with no evident neuroradiological changes [14]. To date, the timing and necessity for the administration of bevacizumab therapy have been topics of debate due to conflicting data available and the possible side effects associated with its use. Now, in our patient, a “wait-and-see approach” was preferred in light of a spontaneous slight improvement from the neuroradiological point of view and the positive neuropsychomotor evolution.

## 5. Conclusions

Clinical data do not typically correlate with radiological findings, rendering clinical-radiological discrepancy an important clue to diagnosing LCC. However, this peculiarity makes clinical follow-up without associated radiological examinations an unreliable tool for disease severity prediction. The mild clinical picture without signs of neurological degeneration associated with an early diagnosis with a clinical-radiological follow-up showing spontaneous amelioration in some early neuroradiological features of the disease are relevant characteristics found in our patient, which can lead to a better understanding of the natural history of LCC. Regarding our patient, we plan to continue the clinical and neuroradiological follow-up (with a “quick” MRI protocol to avoid sedation) to obtain further elements on the spontaneous natural history of the disease. Moreover, it appears very important to set multi-centric collaborative projects to gather clinical descriptions of more patients with LCC and their follow-up standardized evaluations. This is of uttermost importance concerning finding clear genotype-phenotype correlations, if present, and to further understanding how radiological and clinical data during VEGF treatment should be interpreted in light of plausible spontaneous positive changes both in clinical and radiological outcomes.

## Figures and Tables

**Table 1:** Literature overview of pediatric-onset LCC cases.

Patient	Sex	*SNORD118* Variants	Age at Presentation	Presenting Features	Age at First Brain Imaging	Surgery	Therapy with Bevacizumab	Follow-Up	Age at Last Evaluation/Death
Case 3 in Iwama et al., 2016 [18]	F	n.39G > C/n.103G > A	11 mo	S	11 mo	Yes, at 2 y and 10 mo, for RICP due to cystic enlargement with a good outcome.	No	Yes	>2 y and 10 mo
Case 5 in Iwama et al., 2016 [18]	M	n.39G > C/n.72A > G	1 mo	S	1 mo	Yes (from 6 y.o. onward), four surgeries for RICP due to cystic enlargement with partial results	No	Yes	8 y.o.
Case 6 in Iwama et al., 2016 [18]	M	n.3C > T/n.24C > T	9 y.o.	MD	2 y.o. as a control for neonatal meningitis showing the typical alterations of LCC	No	No	Yes	9 y.o.
Only patient in Fay et al., 2017 [19]	M	n.*5C > G/n. 81G > A	11 wks	S	6 y.o.	No	Yes (at 18 y.o.), with both clinical and radiological improvement	Yes	19 y.o.
Only patient in Iwasaki et al., 2017 [20]	F	c.38C > G/c.116G > C	4 y.o.	S	37 y.o.	No	No	Yes	61 y.o.
Patient 1 in Hermens et al., 2018 [21]	M	n.24C > A/n.82A > G	11 mo	S	9 y.o.	No	No	No	9 y.o.
Patient 2 (sister of patient 1) in Hermens et al., 2018 [21]	F	n.24C > A/n.82A > G	4 y.o.	DD	4 y.o.	No	No	No	4 y.o.
Only patient in Taglia et al., 2018 [22]	M	n.57G > A/n.*10G > T	2 mo	S	11 y.o.	No	No	No	23 y.o.
Only patient in Shtaya et al., 2019 [16]	M	n.72A > G/n.*10G > T	12 y.o.	RICP	12 y.o.	Yes (at 12 y.o.), once for RICP with clinical improvement for twelve months	No	Yes	13 y.o.
Patient 3 in Osman et al., 2019 [3]	F	n.72A > G/n.92C > T	11 y.o.	S	11 y.o.	Yes (from 5 y.o. onward), multiple times for RICP due to cystic enlargement	No	Yes	30 y.o., deceased
Only patient in Holland et al., 2020 [23]	M	n.*10G > T/n.24C > T	10 y.o. (CH from 8 y.o.)	S	NA	Yes (he received the third surgery at 20 y.o., age at first surgery not reported), multiple times for RICP due to cystic enlargement	No	Yes	20 y.o.
Only patient in Martinez-Matilla et al., 2020 [24]	M	homozygous n.8G > C	19 mo	MD	19 mo	No	Yes (at 3 years and 4 months), with only radiological improvement	Yes	3 y.o.
F172 in Crow et al., 2020 [1]	M	n.56dup/n.*10G > T	8 wks	S	NA	NA	NA	NA	19 y.o.
F278 in Crow et al., 2020 [1]	M	n.8G > C/n.75A > G	1 y.o.	S	NA	NA	NA	NA	16 y.o., deceased
F281 in Crow et al., 2020 [1]	M	n.3C > T/n.81G > C	4 y.o.	MD	NA	NA	NA	NA	15 y.o.
F285 in Crow et al., 2020 [1]	M	n.57G > A/n.*5C > G	12 y.o.	S	NA	NA	NA	NA	32 y.o., deceased
F309 in Crow et al., 2020 [1]	F	n.3C > T/n.131C > G	2 y.o.	DD	NA	NA	NA	NA	26 y.o.
F330 in Crow et al., 2020 [1]	M	n.8G > A/n.82A > G	3 y.o.	MD	NA	NA	NA	NA	22 y.o.
F331.1 in Crow et al., 2020 [1]	F	n.72A > G/n.*10G > T	2 y.o.	MD	NA	NA	NA	NA	25 y.o.
F331.2 in Crow et al., 2020 [1]	F	n.72A > G/n.*10G > T	5 mo	S	NA	NA	NA	NA	15 y.o., deceased
F334 in Crow et al., 2020 [1]	F	n.82A > G/n.*10G > T	6 y.o.	DD	NA	NA	NA	NA	30 y.o.
F337 in Crow et al., 2020 [1]	M	n.2T > C/n.58dup	18 mo	S	NA	NA	NA	NA	25 y.o.
F343 in Crow et al., 2020 [1]	M	n.-7_22dup/n.*5C > G	9 y.o.	MD	NA	NA	NA	NA	38 y.o.
F344 in Crow et al., 2020 [1]	M	n.8G > C homozygous	7 wks	S	NA	NA	NA	NA	14 y.o.
F362.1 in Crow et al., 2020 [1]	F	n.20C > T/n.*5C > G	2 y.o.	S	NA	NA	NA	NA	15 y.o.
F362.2 in Crow et al., 2020 [1]	F	n.20C > T/n.*5C > G	2 y.o.	DD	NA	NA	NA	NA	13 y.o.
F362.3 in Crow et al., 2020 [1]	M	n.20C > T/n.*5C > G	2 y.o.	DD	NA	NA	NA	NA	9 y.o.
F426.1 in Crow et al., 2020 [1]	M	n.81G > A/n.*5C > G	<6 mo	DD	NA	NA	NA	NA	24 y.o.
F426.2 in Crow et al., 2020 [1]	M	n.81G > A/n.*5C > G	<6 mo	DD	NA	NA	NA	NA	25 y.o.
F445 in Crow et al., 2020 [1]	F	n.127C > G/n.*5C > G	6 mo	S	NA	NA	NA	NA	11 y.o.
F446 in Crow et al., 2020 [1]	M	n.*5C > G homozygous	10 mo	S	NA	NA	NA	NA	13 y.o., deceased
F454.1 in Crow et al., 2020 [1]	F	n.-54_-49del/n.*5C > G	14 y.o.	MD	NA	NA	NA	NA	15 y.o.
F454.2 in Crow et al., 2020 [1]	F	n.-54_-49del/n.*5C > G	11 y.o.	MD	NA	NA	NA	NA	12 y.o.
F465 in Crow et al., 2020 [1]	M	n.60_61insT/n.82A > G	15 mo	S	NA	NA	NA	NA	13 y.o.
F521.1 in Crow et al., 2020 [1]	F	n.104G > A/n.131C > G	<12 mo	DD	NA	NA	NA	NA	18 y.o.
F521.2 in Crow et al., 2020 [1]	M	n.104G > A/n.131C > G	1 y.o.	S	NA	NA	NA	NA	14 y.o.
F551 in Crow et al., 2020 [1]	F	n.127C > G/n.*9C > T	<1 y.o.	DD	NA	NA	NA	NA	28 y.o., deceased
F564 in Crow et al., 2020 [1]	M	n.126C > T/n.*1C > T	9 mo	DD	NA	NA	NA	NA	11 y.o.
F641 in Crow et al., 2020 [1]	M	n.61A > G/n.*10G > T	4 mo	DD	NA	NA	NA	NA	10 y.o.
F691 in Crow et al., 2020 [1]	M	n.58A > G/n.*9C > T	10 mo	S	NA	NA	NA	NA	9 y.o.
F730 in Crow et al., 2020 [1]	F	n.81G > A/n.*10G > T	2 mo	S	NA	NA	NA	NA	11 y.o.
F766 in Crow et al., 2020 [1]	F	n.3C > A/n.42G > A	12 y.o.	MD	NA	NA	NA	NA	16 y.o.
F780.1 in Crow et al., 2020 [1]	F	n.131C > G/n.*1C > T	3 mo	S	NA	NA	NA	NA	19 y.o.
F780.2 in Crow et al., 2020 [1]	F	n.131C > G/n.*1C > T	6 y.o.	DD	NA	NA	NA	NA	11 y.o.
F819.1 in Crow et al., 2020 [1]	F	n.-70_n.?*76del/n.*1C > T	12 y.o.	S	NA	NA	NA	NA	37 y.o.
F819.2 in Crow et al., 2020 [1]	M	n.-70_n.?*76del/n.*9C > T	5 y.o.	DD	NA	NA	NA	NA	36 y.o., deceased
F856 in Crow et al., 2020 [1]	M	n.24C > T/n.*10G > T	11 y.o.	RICP	NA	NA	NA	NA	21 y.o.
F906 in Crow et al., 2020 [1]	M	n.39G > C/n.103G > A	1 y.o.	DD	NA	NA	NA	NA	10 y.o.
F1127 in Crow et al., 2020 [1]	M	n.100T > G/n.*9C > T	<6 mo	S	NA	NA	NA	NA	7 y.o.
F1288 in Crow et al., 2020 [1]	F	n.59T > G/n.*9C > T	3 y.o.	MD	NA	NA	NA	NA	20 y.o.
F1424 in Crow et al., 2020 [1]	F	n.-6G > A/n.130T > C	6 mo	S	NA	NA	NA	NA	13 y.o.
F1445 in Crow et al., 2020 [1]	F	n.3C > T/n.81G > A	8 y.o.	MD	NA	NA	NA	NA	12 y.o., deceased
F1954 in Crow et al., 2020 [1]	M	n.39G > Thomozygous	4 mo	S	NA	NA	NA	NA	15 y.o.
F2054 in Crow et al., 2020 [1]	M	n.20C > T/n.*10G > T	<6 mo	S	NA	NA	NA	NA	5 y.o.
F2380 in Crow et al., 2020 [1]	F	n.57G > T/n.*5C > G	2 mo	S	NA	NA	NA	NA	6 y.o.
F2480 in Crow et al., 2020 [1]	M	n.72A > G/n.*10G > T	12 y.o.	RICP	NA	NA	NA	NA	14 y.o.
F2494 in Crow et al., 2020 [1]	M	n.3C > T/n.57G > A	12 y.o.	RICP	NA	NA	NA	NA	16 y.o.
F2689 in Crow et al., 2020 [1]	F	n.39G > C/n.75A > C	2 y.o.	MD	NA	NA	NA	NA	35 y.o.
F2737 in Crow et al., 2020 [1]	F	n.56dup/n.*5C > G	3 wks	S	NA	NA	NA	NA	14 y.o.
F2871 in Crow et al., 2020 [1]	F	n.-54_-49del/n.*9C > T	9 y.o.	RICP	NA	NA	NA	NA	10 y.o.
N11301 in Crow et al., 2020 [1]	M	n.3C > A/n.20C > T	15 y.o.	MD	NA	NA	NA	NA	48 y.o.
Hild_1 in Crow et al., 2020 [1]	M	n.8G > C homozygous	1 y.o.	DD	NA	NA	NA	NA	4 y.o.
Patient 6 (only patient in Machnikowska-Sokołowska et al., 2020) [25]	F	n.19C > G/n.*5C > G	10 y.o.	CH	10 y.o.	No	No	Yes	11 y.o.
Only patient in Sim et al., 2021 [26]	F	n.*9C > T and n.24C > T	Childhood	ID	19 y.o.	No	No	No	22 y.o.
Only patient in Jin et al., 2021 [27]	M	n.∗9C > T and n.3C > T	10 days	S	1 mo	No	No	No	4 y and 1 mo
Only patient in Kodama et al., 2022 [4]	F	n.41G > T/n.90C > T	3 mo	S	3 mo	No	No	Yes	4 y and 8 mo
Only patient in Li et al., 2022 [28]	M	n.*6C > T/n.70G > A	16 y.o.	S	55 y.o.	No	No	No	55 y.o.

Abbreviations: CH, cronic headache; DD, developmental delay; ID, intellectual disability; F, female; mo, months; M, male; MD, motor disorder; NA, not available; RICP, raised intracranial pressure; S, seizures; wks, weeks; y.o., years old.

## Data Availability

The data presented in this study are available on request from the corresponding author. The data are not publicly available due to privacy.
